# Allantoin Serves as a Novel Risk Factor for the Progression of MASLD

**DOI:** 10.3390/antiox14050500

**Published:** 2025-04-22

**Authors:** Weiqiang Lv, Xueqiang Wang, Zhaode Feng, Cunxiao Sun, Hansen Wu, Mengqi Zeng, Tianlin Gao, Ke Cao, Jie Xu, Xuan Zou, Tielin Yang, Hao Li, Lei Chen, Jiankang Liu, Shanshan Dong, Zhihui Feng

**Affiliations:** 1 Center for Mitochondrial Biology and Medicine, The Key Laboratory of Biomedical Information Engineering of Ministry of Education, School of Life Science and Technology, Xi’an Jiaotong University, Xi’an 710049, China; gl824705609@stu.xjtu.edu.cn (W.L.); fengzhaode@stu.xjtu.edu.cn (Z.F.); caoke2016@mail.xjtu.edu.cn (K.C.); xj0322@xjtu.edu.cn (J.X.); lihao@xjtu.edu.cn (H.L.); j.liu@mail.xjtu.edu.cn (J.L.); 2 Frontier Institute of Science and Technology, Xi’an Jiaotong University, Xi’an 710049, China; 4123128024@stu.xjtu.edu.cn (C.S.); whs740211756@outlook.com (H.W.); 3 School of Health and Life Sciences, University of Health and Rehabilitation Sciences, Qingdao 266100, China; wangxueqiang@uhrs.edu.cn (X.W.); zengmengqi@uhrs.edu.cn (M.Z.); chenlei@uhrs.edu.cn (L.C.); 4 School of Public Health, Qingdao University, Qingdao 266071, China; gaotl@qdu.edu.cn; 5 Department of Geriatrics Cardiology, The Second Affiliated Hospital of Xi’an Jiaotong University, Xi’an 710004, China; wooair@gmail.com; 6 Precision Medical Institute, The Second Affiliated Hospital of Xi’an Jiaotong University, Xi’an 710004, China; 7 Biomedical Informatics & Genomics Center, The Key Laboratory of Biomedical Information Engineering of Ministry of Education, School of Life Science and Technology, Xi’an Jiaotong University, Xi’an 710049, China; yangtielin@xjtu.edu.cn (T.Y.); 8 Interdisciplinary Research Center of Frontier Science and Technology, Xi’an Jiaotong University, Xi’an 710049, China

**Keywords:** MASLD, mendelian randomization, allantoin, uric acid, PPARα

## Abstract

Uric acid (UA), traditionally recognized as an extracellular antioxidant, exhibits paradoxical associations with metabolic disorders such as metabolic dysfunction-associated steatotic liver disease (MASLD), though its mechanistic contributions remain elusive. Here, we integrate multi-modal evidence to explore the role of UA and its oxidative metabolite, allantoin, in MASLD progression. Analysis of UK Biobank data revealed a strong association between elevated UA levels and increased risks of MASLD and type 2 diabetes (T2D). However, Mendelian randomization analysis of over 2 million samples demonstrated causal effects of urate solely on serum triglycerides and T2D risk. Targeted metabolomics in an elderly Chinese cohort identified allantoin, an oxidative by-product of UA, significantly elevated in individuals with dyslipidemia or T2D, with serum allantoin levels positively correlated with fasting glucose, triglycerides, and cholesterol. Animal studies indicated that allantoin exacerbates hepatic lipid accumulation and glucose intolerance in high-fat diet mice, driven by increased hepatic lipid biogenesis and reduced bile acid production. Notably, further research revealed a strong binding affinity of allantoin for PPARα, leading to the suppression of PPARα activity, which promotes the progression of MASLD. These findings underscore the critical role of allantoin, rather than UA, as a critical driver of MASLD development, offering valuable insights for the prediction and management of hepatic metabolic disorders.

## 1. Introduction

Metabolic dysfunction-associated steatotic liver disease (MASLD), the recently proposed nomenclature replacing non-alcoholic fatty liver disease (NAFLD), has emerged as a leading cause of liver-related mortality and morbidity [1,2]. The condition is closely associated with metabolic syndrome, including obesity, type 2 diabetes (T2D), and dyslipidemia (DLP), all characterized by disruptions in glucose and lipid metabolism [3,4]. Notably, 69% of MASLD patients present with DLP, and 22% had T2D, highlighting the central role of lipid metabolism imbalance in disease progression [5]. While triglycerides (TG) constitute the predominant lipid type accumulating in the liver, emerging evidence also underscores cholesterol accumulation as a critical contributor to MASLD progression [6,7]. Despite ongoing efforts, the mechanistic interplay of lipid metabolism remains incompletely understood, necessitating further exploration of novel risk factors and therapeutic targets.

Uric acid (UA), the terminal product of purine metabolism, is further converted to allantoin in most mammals, except humans, who lose the function of uricase during evolution [8]. Though UA serves as a major plasma antioxidant, it paradoxically correlates with oxidative stress-associated pathologies [9]. Beyond its established role in gout and kidney disease, hyperuricemia is increasingly implicated in lipid dysregulation. A 5-year cohort study conducted in Japan revealed an increased risk of low-density lipoprotein (LDL) and hypertriglyceridemia with elevated serum UA levels [10]. Similarly, analysis of National Health and Nutrition Examination Survey (NHANES) data (2007–2018) demonstrated a significant positive association between serum UA level and hypertriglyceridemia in US adults [11]. A 5-year follow-up study involving 9837 non-obese Chinese participants further revealed that serum UA to HDL-cholesterol ratio independently predicts MASLD incidence [12]. Preclinical studies corroborate these findings, showing UA exacerbates hepatic lipid accumulation, insulin resistance, and DLP in mice [13,14]. Nevertheless, the detailed mechanisms linking UA to lipid metabolism remain elusive.

Interestingly, despite the lack of uricase activity in humans, UA can still be non-enzymatically oxidized into allantoin by reactive oxygen species (ROS) [15], promoting the development of analytical methods to quantify allantoin in body fluids [16,17]. While allantoin is widely used as an oxidative stress marker [18,19], its pathophysiological roles remain controversial. Previous studies have suggested that allantoin may have benefits on wound healing and ovalbumin-induced lung inflammation [20,21], while Yang et al. reported that allantoin could induce pruritus in chronic kidney disease [22]. Metabolomic profiling identified allantoin as one of the four major metabolites that significantly increased in gestational diabetes mellitus women [23], and murine studies associate urinary allantoin with atherosclerosis progression [24]. Conversely, diabetes rats with MASLD exhibited reduced urinary allantoin levels correlating with DLP and hepatic dysfunction [25]. These contradictory findings imply a context-dependent role for allantoin in metabolic regulation. In the present study, we establish allantoin as the key mediator linking UA to MASLD-related metabolic disorders. Under oxidative stress conditions, UA oxidation drives allantoin elevation, which in turn exacerbates hepatic lipid deposition by modulating TG and bile acid synthesis. Our findings position allantoin, not UA, as the central driver of MASLD progression, offering novel mechanistic insights and therapeutic avenues for metabolic liver diseases.

## 2. Materials and Methods

### 2.1. Antibodies and Reagents

Antibodies against ACC (3676), p-ACC (3661), and GAPDH (5174) were purchased from Cell Signaling Technology (Danvers, MA, USA). Antibody against FASN (55580) was purchased from Santa Cruz Biotechnology (Santa Cruz, CA, USA). Allantoin (05670) was purchased from Sigma (St. Louis, MO, USA). GW6471(S2798), WY14643 (S8029), and rilmenidine (S7548) were purchased from Selleck (Shanghai, China). Nuclear Extraction Kit (113474), PPAR alpha Transcription Factor Assay Kit (133107), and NBD Cholesterol Staining Dye Kit (269448) were purchased from Abcam (Cambridge, UK). Other reagents used in this study were purchased from Sigma (St. Louis, MO, USA).

### 2.2. Human Subjects

The case-control study was approved by the Ethics Committee of Qingdao University School of Medicine (QDU-HEC-2022282). All subjects signed an informed consent document before enrolling in the study. A total of 320 participants, including T2D (126), DLP (89), and age-matched health control (105) individuals, were recruited from 2021 to 2023 at the Health-center of Licha Town, Jiaozhou City, Qingdao, China. Diabetes was defined as HbA1c ≥ 6.5%, fasting blood glucose (FBG) ≥ 7.0 mmol/L, or 2 h oral glucose tolerance (OGTT) ≥ 11.1 mmol/L according to the guideline for the prevention and treatment of T2D in China (2020 edition). DLP was defined as triglycerides (TG) ≥ 2.3 mmol/L, or TC ≥ 6.2 mmol/L according to the guideline for management of DLP in China (2023 edition).

### 2.3. Individual-Level Data from UK Biobank

**Figure 1:**
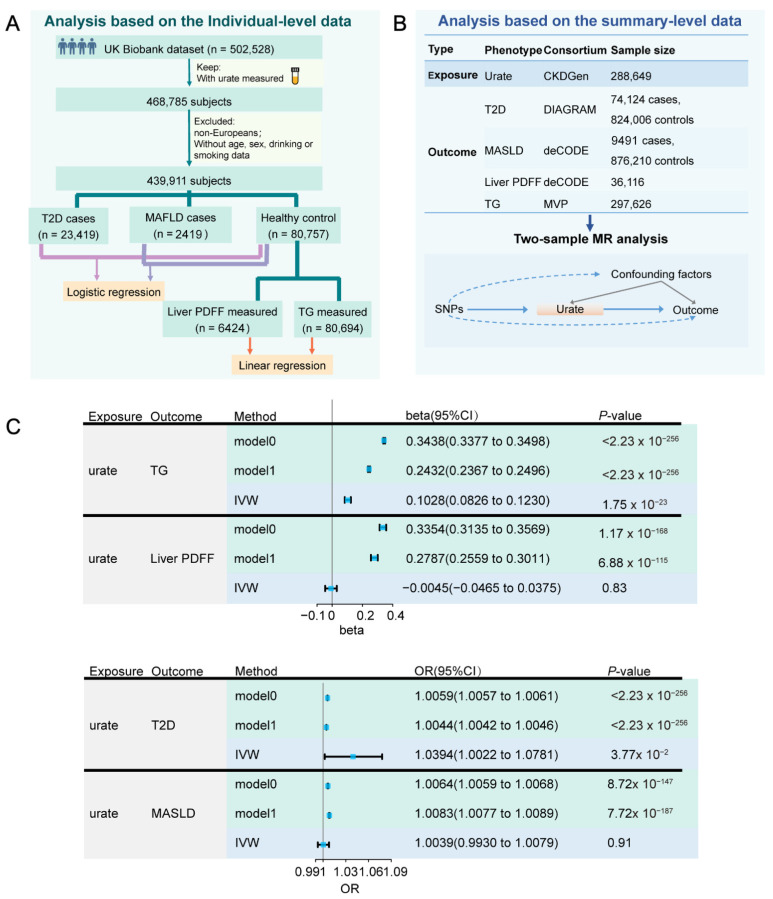
Assessment of the relationships between urate and other traits (TG, liver PDFF, T2D, and MASLD). Both individual-level data and summary-level data were used. (**A**) The sample selection process of the individual-level data. (**B**) Information of the summary level data for two-sample Mendelian randomization (MR) analysis. (**C**) Summary of effect estimates from the different methods for the urate on T2D, MASLD, TG, and liver PDFF. For individual-level data analysis (with light green as the background color), model0 refers to analysis without covariates, and model1 refers to analysis with sex, age, smoking, and drinking status as covariates). For summary-level data analysis (with light blue as the background color), the results of the primary method (inverse-variance weighted, (IVW)) are shown. TG: triglycerides; T2D: type 2 diabetes; MASLD: non-alcoholic fatty liver disease; liver PDFF: magnetic resonance imaging derived liver proton density fat fraction.

The individual-level data were obtained from the UK Biobank (application ID 46387). All subjects provided written informed consent. We obtained phenotype data from 502,528 participants. The detailed field ID or International Classification of Diseases (ICD) code for each trait is shown in Supplementary Table S1. In addition, we considered age, sex, smoking, and drinking status as confounders. A total of 468,785 subjects took the urate test. As a quality control, non-European participants and those without confounder information were excluded. Among the remaining subjects (n = 439,911), the number of T2D and MASLD patients is 23,419 and 2419, respectively. 92,246 individuals are healthy controls with no disease record in the ICD-10 of the UK Biobank. Among these controls, the number of subjects with measured TG and magnetic resonance imaging derived liver proton density fat fraction (PDFF) are 80,694 and 6424, respectively. The data filtering process is shown in Figure 1A.

### 2.4. Observational Analysis

Using the individual-level data from the UK Biobank, we performed linear regression to estimate the relationship between TG/liver PDFF and urate. Logistic regression was used to assess the relationship between T2D/MASLD and urate. We used two models: model0 (without covariate), and model1 (including sex, age, smoking, and drinking status as covariates).

### 2.5. GWAS Summary Data

For the exposure, GWAS summary statistics of urate with a sample size of 288,649 individuals of European ancestry were obtained from the CKDGen consortium [26]. For the outcomes, GWAS summary data for T2D were obtained from the DIAGRAM consortium (case number = 74,124, control number = 824,006) [27]. Summary statistics of liver PDFF (n = 36,116) and MASLD (case number = 9491, control number = 876,210) were obtained from the deCODE genetics [28]. Summary statistics of TG were obtained from the Million Veteran Program (MVP) based on (n = 297,626) [29]. Information on these datasets is provided in Figure 1B and Supplementary Table S2. We manually checked the cohorts involved in these datasets and samples in these outcomes were not overlapped with those in the exposure. Before MR analysis, we removed SNPs with minor allele frequencies less than 0.01 in the GWAS datasets. For T2D and MASLD GWAS summary statistics, the OR value was converted to log odds. We removed SNPs located within regions of long-range, high-linkage disequilibrium, such as the human major histocompatibility complex region. The list of these regions was obtained from the following link: https://genome.sph.umich.edu/wiki/Regions_of_high_linkage_disequilibrium_(LD)#cite_note-3 (accessed on 11 March 2024) [30].

### 2.6. Animals

The study for human samples collection was conducted in accordance with the Declaration of Helsinki, and approved by the Institutional Review Board of Qingdao University School of Medicine (QDU-HEC-2022282; 12 February 2022). The animal study protocol was approved by the Institutional Animal Care and Use Committee of Xi’an Jiaotong University (No. 2018-0026; 22 October 2018). The mice were maintained in a specific-pathogen-free (SPF) environment under a 12 h light/12 h dark cycle at 23–25 °C with 60% ± 10% relative humidity. For the diet-induced metabolic stress mouse model, a chow diet (control, 10% kcal fat content, D12492, Research Diets, New Brunswick, NJ, USA) and a high-fat diet (HFD, 60% kcal fat content, D12450, Research Diets, New Brunswick, NJ, USA) were used. For allantoin intervention, mice were fed on either a chow diet or HFD for 4 weeks, the HFD-fed mice were then supplemented with allantoin in their drinking water at the concentrations of 0.06 mg/mL and 0.3 mg/mL for an additional 4 weeks. This study included four groups: mice feeding chow diet (Chow), mice feeding HFD (HFD), mice feeding HFD with allantoin supplement at 0.06 mg/mL (HFD + Allantoin-L), and mice feeding HFD with allantoin supplement at 0.3 mg/mL (HFD + Allantoin-H). A total of forty C57BL/6 J male mice (8 weeks old) were randomly assigned to these four groups. A glucose tolerance test and insulin tolerance test were conducted at the end of the treatment period.

### 2.7. Recombinant Proteins

The cDNA of homo and murine PPARα was cloned into the pET28a-6xHis *E. coli* expression vector to create a fusion protein with an N-terminal His tag. For in vivo expression, *E. coli* BL21 (DE3) cells transformed with the plasmid were cultured in an LB medium containing 100 μg/mL kanamycin at 37 °C until the OD 600 reached 0.6–0.8. Induction was then initiated with 1 mM IPTG at 18 °C for 16 h. Following induction, the bacteria were harvested by centrifugation at 12,000× *g* and 4 °C and then resuspended in lysis buffer (20 mM Tris, 150 mM NaCl, pH 8.0) prior to sonication on ice. The supernatant containing the target proteins was collected by centrifugation (15,000× *g*, 4 °C), and purified using an ÄKTA purification system (GE Healthcare, Princeton, NJ, USA) equipped with a HisTrap HP Ni Sepharose column (Cytiva, 17524801, Shanghai, China). Column-bound proteins were washed with buffer (20 mM Tris, 150 mM NaCl, pH 8.0) and eluted using a buffer containing 20 mM Tris, 150 mM NaCl, and 500 mM imidazole (pH 8.0). The eluate was concentrated to approximately 1 mL by ultrafiltration (Amicon Ultra 15 mL, 10 kDa, UFC9010) to remove nonspecifically bound proteins, and then further purified by injecting it into a Superdex 75 10/300 Exclusion Chromatography column (Cytiva, 29148721, Shanghai, China) using upwelling buffer (20 mM Tris, 150 mM NaCl, pH 8.0). The purified proteins were concentrated by ultrafiltration with 10% glycerol and stored at −80 °C for future tests.

### 2.8. Dual-Luciferase Reporter Assay

The peroxisome proliferator-activated receptor alpha response element (PPRE) was cloned upstream of the firefly luciferase gene in the pGL4-TA-Luc vector. The pGL4 basic and pRL-TK plasmids were purchased from Promega (Beijing, China). Hep3B cells were transfected at 60% confluency in 12-well plates with the pGL4 basic plasmid as a negative control, while the remaining wells were transfected with 500 ng of the PPRE-Luc plasmid and 500 ng of the pRL-TK plasmid. After 8 h of transfection, the cells were treated with 10 μM wy14643 and 100 nM or 1 μM allantoin for 36 h. After 48 h of transfection, the cells were collected for luciferase activity measurement following the manufacturer’s protocol. The relative light units (RLUs) from the firefly luciferase readings were adjusted by subtracting the values obtained from the pGL4 basic transfected control. The ratio of firefly luciferase activity to Renilla luciferase activity was calculated for each sample to normalize the data, accounting for variations in transfection efficiency and cell viability. Statistical analysis was conducted using GraphPad Prism (Version 10.2.0) to compare the ratios across different experimental groups.

### 2.9. Surface Plasmon Resonance (SPR)

All SPR measurements were conducted using a four-channel optical biosensor, the Biacore T200 (GE Healthcare, Princeton, NJ, USA), operated through the Biacore T200 Control Software (Version 3.1). To activate the dextran on the surface of the CM5 sensor chip, a mixture of 0.1 M N-hydroxysuccinimide (NHS) and 0.4 M 1-ethyl-3-(3-dimethylaminopropyl) carbodiimide hydrochloride (EDC) was applied for 7 min at a flow rate of 5 μL/min. The PPARα protein (10 μg) was then dissolved in a sodium acetate buffer at pH 4 and immobilized on the dextran surface of the CM5 optical chip via the amino groups of the protein in the working channel (Fc2) at a flow rate of 10 μL/min. The first channel (Fc1), which did not have protein immobilized, served as a control to account for non-specific binding of the protein to the chip surface. To inactivate the chip surface, 1 M ethanolamine (pH 8.5) was injected for 5 min at a flow rate of 10 μL/min. For the surface test, analyte dilutions were prepared in the running buffer at varying concentrations. Three startup cycles were performed with the running buffer to stabilize the baseline signal before initiating the experiment. A manual run was then started with a flow rate set at 10 μL/min, an injection time of 2 min, and a dissociation time of 3 min. A series of analyte dilutions were created based on the results from the surface test, consisting of at least five concentrations along with one or two zero-level controls for double referencing. Upon completion of the experiment, the raw data were processed using Biacore software, which included baseline correction and background subtraction. Affinity constants were derived from the binding curves to evaluate the affinity between the ligand and the analyte.

### 2.10. Statistical Analysis

Data were analyzed with GraphPad Prism-9 software and presented as the mean ± standard error of the mean (SEM). The Gaussian distribution of the data was assessed using Kolmogorov-Smirnov’s and Shapiro-Wilk’s tests. Pairwise comparisons were analyzed using a two-tailed Student’s *t*-test. Correlation analysis was conducted using simple linear regression. Other data were analyzed using one-way ANOVA with Tukey’s multiple comparison test or two-way ANOVA with multiple comparison tests. In all cases, *p* < 0.05 was considered significant.

## 3. Results

### 3.1. Close Association of UA with Risk of MASLD

To elucidate the detailed correlation of UA with MASLD development, we analyzed individual-level data from the UK Biobank (application ID 46387). Given the established role of T2D and DLP as key MASLD risk factors, we incorporated T2D status and TG levels into our analysis. Following a rigorous filtering process, the cohort includes 23,419 T2D cases, 2419 MASLD cases, and 80,757 healthy controls (Figure 1A). Linear regression was employed to estimate the relationship between TG/PDFF and UA in healthy controls, while logistic regression evaluated UA’s association with T2D/MASLD risk. Elevated UA correlated strongly with higher levels of TG, liver PDFF, and higher risk of T2D and MASLD (Figure 1C, model0). Notably, these associations persisted after adjusting potential confounders including sex, age, smoking, and alcohol consumption (Figure 1C, model1). For instance, high UA level was significantly associated with increased TG levels (model0: β = 0.3438, 95% CI 0.3377 to 0.3498, *p* < 2.23 × 10^−308^, model1: β = 0.2432, 95% CI 0.2367 to 0.2496, *p* < 2.23 × 10^−308^).

To further assess the causal effects of UA on T2D, MASLD, TG, and liver PDFF, we conducted a two-sample mendelian randomization (MR) analysis (Figure 1B). Detail information of the instrumental variables (IVs) is provided in Table S3. Our analysis did not reveal significant causal effects of UA on MASLD and liver PDFF (Figure 1C, Figure S1 and Table S4). However, elevated UA level was found to significantly contribute to increased risk of T2D (OR = 1.0394, CI 1.0022 to 1.0781, *p* = 0.0377) and increased TG level (β = 0.1028, 95% CI 0.0826 to 0.1230, *p* = 1.75 × 10^−23^) (Figure 1C and Figure S1), which is consistent with previous finding [31]. Leave-one-out analyses confirmed that no single SNP was responsible for driving the causal estimates (Figure S1E). Moreover, MR-Egger intercepts for these two associations approached zero, suggesting a lack of significant pleiotropy (Table S5). Consistently, the MR-PRESSO global test detected no significant pleiotropy, supporting the validity of our IVs (*p* > 0.05, Table S5).

### 3.2. Serum Allantoin Is Positively Correlated with DLP

**Figure 2:**
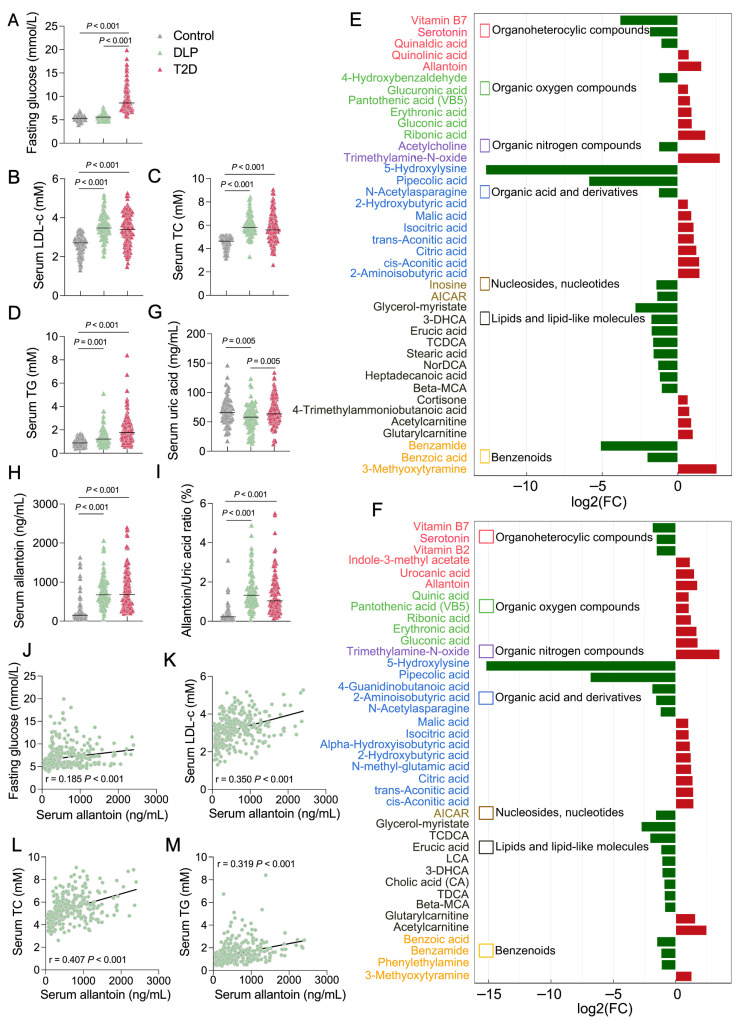
Positive correlation of serum allantoin with DLP in Chinese elders. Comparative analysis of metabolic traits in Chinese elders: (**A**) fasting glucose, (**B**) Serum LDL-c, (**C**) Serum TC, and (**D**) Serum TG (healthy control, n = 105; DLP, n = 89; T2D, n = 126). (**E**) Top 40 significantly changed metabolites in DLP group compared to control. (**F**) Top 40 significantly changed metabolites in T2D group compared to control. (**G**) Serum uric acid level, (**H**) allantoin level, and (**I**) the ratio of allantoin to UA in Chinese elders. Correlation analysis of serum allantoin level with fasting glucose (**J**), serum LDL-c (**K**), serum TC (**L**), and serum TG (**M**) in Chinese elders. Each dot represents one biological replicate. Statistical analysis was conducted using either a two-tailed unpaired *t*-test or simple linear regression. LDL-c, low-density lipoprotein cholesterol; TC, total cholesterol; TG, triglyceride; DLP: dyslipidemia; T2D: type 2 diabetes.

To investigate UA’s relationship with DLP and T2D, we established a human diabetic cohort in Qingdao, China enrolling 320 participants including healthy controls and individuals clinically diagnosed with DLP or T2D. Fasting glucose levels were elevated exclusively in the T2D group (Figure 2A), while elevated cholesterol (TC) and TG levels were noted in both DLP and T2D subjects (Figure 2B–D).

Targeted metabolomics analysis of serum samples demonstrated distinct metabolite clustering patterns in DLP or T2D subjects compared to healthy controls (Figure S2A,B). The differential analysis identified 166 significantly altered metabolites in the DLP group (44 increased, 122 decreased, Figure S2C) and 169 in the T2D group (65 increased, 104 decreased, Figure S2D). Notably, the top 40 significantly altered metabolites exhibited similar trends in both DLP and T2D groups (Figure 2E,F). Intriguingly, UA levels were found relatively lower in the DLP group compared to either controls or T2D individuals (Figure 2G). Conversely, allantoin, a non-enzymatically oxidized product of UA was found dramatically increased in both the DLP and T2D groups compared to healthy controls (Figure 2H). The serum allantoin-to-UA ratio followed this trend, further implicating oxidative UA metabolism in metabolic dysfunction (Figure 2I). Additionally, we observed significant positive correlations between serum allantoin levels and fasting glucose, serum LDL-c, serum TC, and serum TG levels across the cohort (Figure 2J–M). These findings position allantoin as a potential contributor to developing DLP.

### 3.3. Allantoin Increases Susceptibility of Glucose Intolerance in Mice Feeding High-Fat Diet

**Figure 3:**
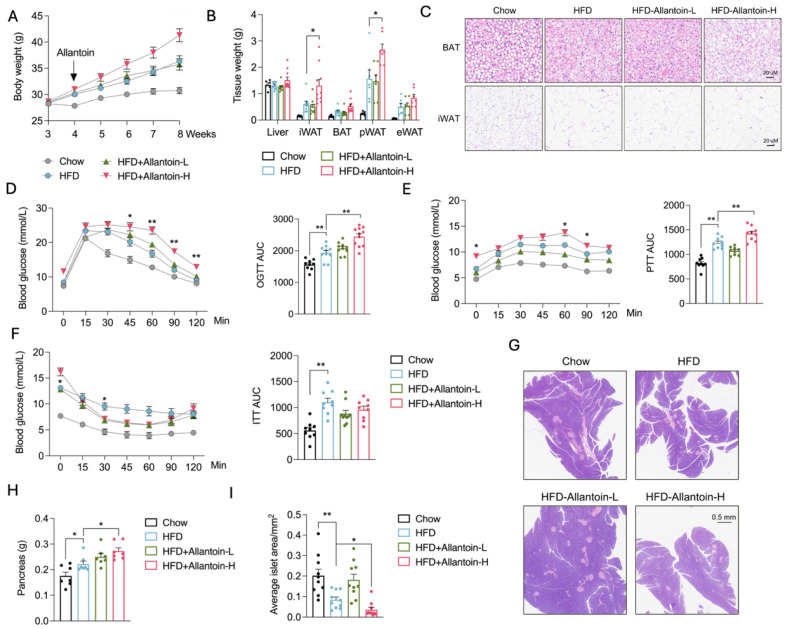
Allantoin increases susceptibility to glucose intolerance in mice feeding high-fat diets. Mice at the age of 8 weeks were subjected to high-fat diet (HFD) for 4 weeks followed by allantoin supplement and HFD feeding for another 4 weeks, metabolic indexes were determined: (**A**) Body weight curve, (**B**) Tissue weight, (**C**) HE staining of BAT and iWAT, (**D**) Glucose tolerance test, (**E**) Pyruvate tolerance test, (**F**) Insulin tolerance test, (**G**) HE staining of pancreas, (**H**) Pancreas weight, and (**I**) analysis of average islet area in four groups. Values are mean ± SEM, n ≥ 8, each dot represents one biological replicate. Statistical analysis was conducted using two-tailed unpaired *t*-test or two-way ANOVA with multiple comparison tests. * *p* < 0.05, ** *p* < 0.01.

To further investigate the effects of allantoin on the development of metabolic disorders, mice fed on a chow diet were administrated with allantoin via drinking water at 60 μg/mL and 300 μg/mL for up to six months. The mice in all three groups showed comparative body weight gain and water intake (Figure S3A,B). Glucose tolerance tests conducted at one, two, and four months of intervention showed consistent comparable changes (Figure S3C–E). However, after six months of high-dose allantoin supplement, mild glucose intolerance emerged (Figure S3F), suggesting that prolonged allantoin exposure may impair metabolic homeostasis under baseline conditions. Subsequently, a high-fat diet (HFD) feeding was exercised in mice with an additional allantoin supplement at the same dose. Notably, the addition of a higher dose of allantoin dramatically increased the body weight gain in mice on the HFD (Figure 3A), primarily attributed to increased mass of white adipose tissues (WAT) (Figure 3B). Additionally, brown adipocytes whitening was evident following high-dose allantoin supplement, while white adipocyte size remained comparable under HFD feeding (Figure 3C), suggesting that increased WAT mass was primarily attributed to increased adipogenesis rather than hypertrophy. Further assessments of glucose and pyruvate tolerance tests indicated impaired glucose metabolism after the high-dose allantoin supplement in HFD-fed mice (Figure 3D,E), paralleled by reduced insulin sensitivity (Figure 3F). Notably, these mice exhibited increased pancreas weight and decreased islet numbers (Figure 3G–I), corroborating the rise in fasting glucose levels (Figure 3F). These findings demonstrate a compelling effect of allantoin in exacerbating glucose intolerance under conditions of metabolic stress.

### 3.4. Allantoin Aggravates Hepatic Lipid Accumulation in Mice Feeding HFD

**Figure 4:**
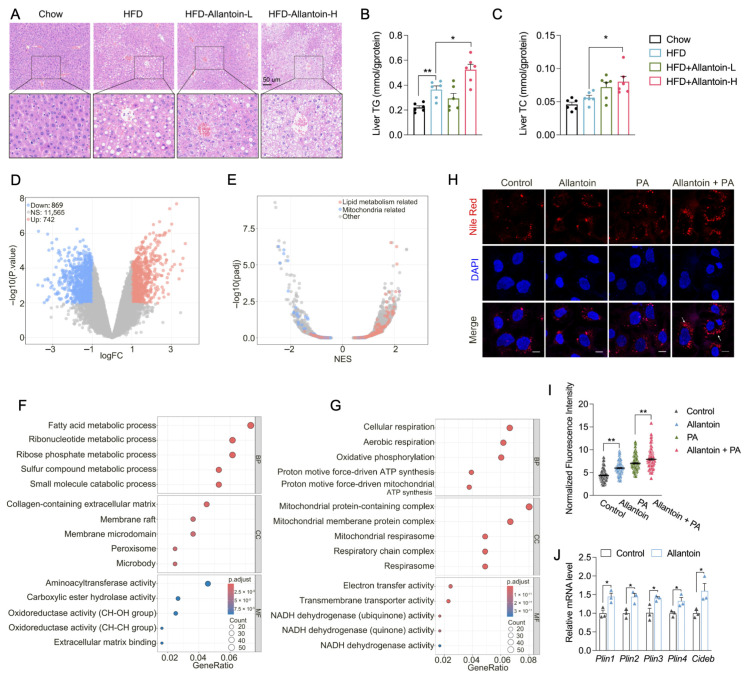
Allantoin aggravates hepatic lipid accumulation in mice feeding high-fat diet. Mice at the age of 8 weeks were subjected to high-fat diet (HFD) for 4 weeks followed by allantoin supplement and HFD feeding for another 4 weeks, hepatic lipid levels were determined: (**A**) HE staining of liver section, (**B**) liver TG level, and (**C**) liver TC level. RNA-seq was performed with liver tissue of HFD-Allantoin-H group and HFD group, (**D**) volcano plot of differentially expressed genes, (**E**) gene set enrichment analysis, (**F**) top 5 upregulated GO term, and (**G**) top 5 downregulated GO term was analyzed. Hep3B cells were pretreated with allantoin at 1 μM for 12 h followed by palmitate treatment at 100 μM for 24 h, nile red staining was performed for lipid visualization (**H**) and quantification (**I**). (**J**) mRNA level of lipid droplets biogenesis related genes in Hep3B cells following allantoin supplement for 12 h. Values are mean ± SEM, n ≥ 3, each dot represents one biological replicate. Statistical analysis was conducted using two-tailed unpaired *t*-test. * *p* < 0.05, ** *p* < 0.01.

In addition to glucose intolerance, high-dose allantoin supplements in HFD-fed mice elevated serum TC levels without altering TG levels (Figure S4A,B). The staining of liver sections consistently indicated enhanced lipid accumulation in Allantoin-H mice, which was further supported by analysis of liver TC and TG contents (Figure 4A–C). RNA-seq analysis of liver tissues identified distinct transcriptional profiles between the HFD and Allantoin-H group (Figure 4D). Gene set enrichment analysis showed marked suppression of mitochondria-related genes and an increase in lipid metabolism-related genes in Allantoin-H mice (Figure 4E). Further GO term analysis identified fatty acid metabolic processes as the most upregulated biological process (Figure 4F), while multiple mitochondrial respiration processes ranked among the top five downregulated biological processes (Figure 4G). qPCR analysis validated the increased expression of lipogenesis and lipid droplet biogenesis genes in Allantoin-H mice (Figure S4C–F). To further verify the effect of allantoin on hepatic lipid accumulation, Hep3B cells were cultured with the addition of allantoin and palmitic acid, Nile red staining revealed that allantoin supplements not only can directly increase the hepatic TG level but also aggravate free fatty acid-induced lipid accumulation in vitro (Figure 4H,I). Consistent with in vivo findings, allantoin upregulated the expression of lipid droplet biogenesis-related genes in hepatocytes (Figure 4J). These findings establish allantoin as a critical driver of MASLD, particularly under conditions of metabolic stress.

### 3.5. Allantoin Suppresses Hepatic Cholesterol Metabolism

**Figure 5:**
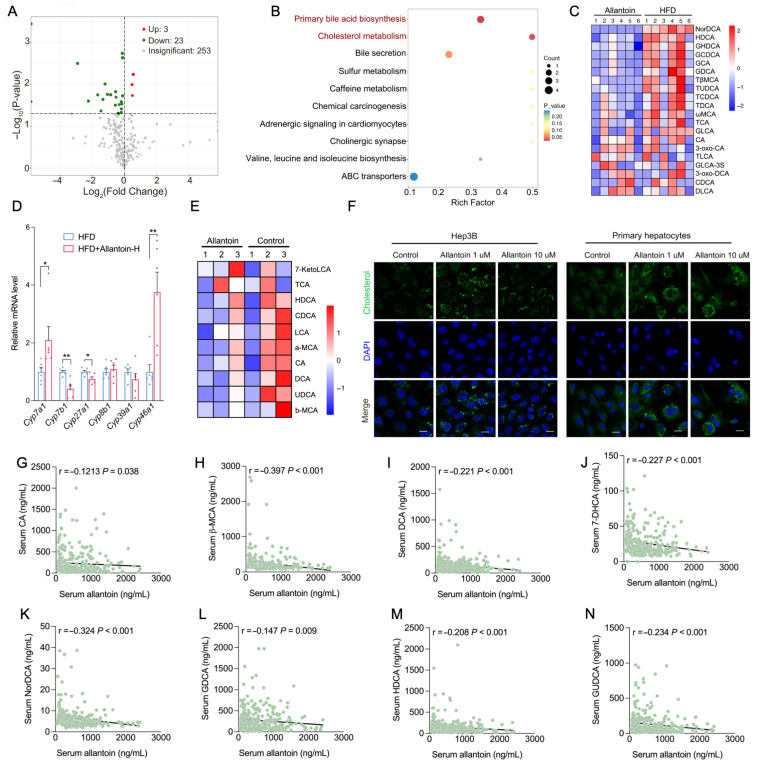
Allantoin suppresses hepatic cholesterol metabolism. Mice were subjected to high-fat diet (HFD) for 4 weeks followed by allantoin supplement and HFD feeding for another 4 weeks, targeted metabolomics with liver tissues were performed: (**A**) volcano plot of significantly changed metabolites, (**B**) KEGG enrichment analysis, and (**C**) heatmap revealing level of all identified bile acids (n = 6). (**D**) Relative mRNA level of bile acid synthesis-related genes in the liver of treated mice (n = 6). (**E**) Heatmap revealing level of identified bile acids in primary hepatocytes after allantoin treatment at 1 μM for 24 h (n = 3). (**F**) Cholesterol staining in Hep3B cells and primary hepatocytes after allantoin treatment for 24 h (Scale bar, 20 μm). Correlation analysis of serum allantoin level with serum bile acids including CA (**G**), DCA (**H**), 7-DHCA (**I**), NorDCA (**J**), HDCA (**K**), GDCA (**L**), GHDCA (**M**), GUDCA (**N**) in Chinese elders. Values are mean ± SEM, each dot represents one biological replicate. Statistical analysis was conducted using either two-tailed unpaired *t*-test or simple linear regression. * *p* < 0.05, ** *p* < 0.01. CA, cholic acid; DCA, deoxycholic acid; 7-DHCA, 7-dehydrocholic acid; NorDCA, 23-nordeoxycholic acid; GDCA, glycodeoxycholic acid; HDCA, hyodeoxycholic acid; GHDCA, glycohyodeoxycholic acid; GUDCA, glycodehydrocholic acid.

To investigate the hepatic metabolic alterations induced by allantoin in the context of HFD, we conducted a targeted metabolomics analysis with mice livers from HFD and Allantoin-H group. Among 279 identified metabolites, 26 exhibited significant changes, with 23 downregulated and 3 upregulated (Figure 5A). KEGG pathway analysis highlights primary bile acid biosynthesis and cholesterol metabolism as the most affected processes (Figure 5B). Notably, most of the identified bile acids were found decreased in the Allantoin-H group (Figure 5C), which was further supported by altered gene expression related to bile acid biogenesis (Figure 5D). Consistently, bile acid profiling in Hep3B cells demonstrated a reduction in bile acid level following allantoin treatment (Figure 5E), while NBD staining revealed significant cholesterol accumulation within both Hep3B cells and primary hepatocytes (Figure 5F), suggesting an impaired synthesis of bile acid from cholesterol. Additionally, targeted metabolomics analysis of enrolled human participants identified significant reductions in primary and secondary bile acids in individuals with DLP and/or T2D compared to healthy controls, including cholic acid (CA), deoxycholic acid (DCA), hyodeoxycholic acid (HDCA), 7-dehydrocholic acid (7-HDCA), taurodeoxycholic acid (TDCA), 3-nordeoxycholic acid (NorDCA), glycodehydrocholic acid (GUDCA), and taurodeoxycholic acid (TDCA) (Figure S5). Furthermore, significant negative correlations were observed between serum allantoin levels and various bile acid levels in these participants (Figure 5G–N). In conjunction with findings showing a positive correlation between serum allantoin and serum TC and LDL-c levels in human subjects (Figure 2J,K), as well as increased serum and liver TC levels in mice following allantoin supplementation (Figure 4C and Figure S4B), we speculate that allantoin may exacerbate cholesterol accumulation by suppressing bile acid biogenesis during the progression of MASLD.

### 3.6. Allantoin Is Potential Endogenous PPARα Antagonist

It is well established that PPARα serves as a master transcriptional regulator in lipid and cholesterol metabolism, both directly or indirectly [32,33], and has been proposed as a therapeutic target for lipid metabolic disorders [34]. Consistent with prior studies, inhibition of PPARα activity promoted expression of lipid biogenesis-related genes and altered expression of bile acid synthesis-related genes in both Hep3B cells (Figure S6A) and primary hepatocytes (Figure S6B), accompanied by marked elevated lipid accumulation in these cells (Figure S6C). These findings align with the observed effects of allantoin supplements on cultured cells and HFD-feeding mice, leading us to hypothesize that allantoin may modulate PPARα activity.

**Figure 6:**
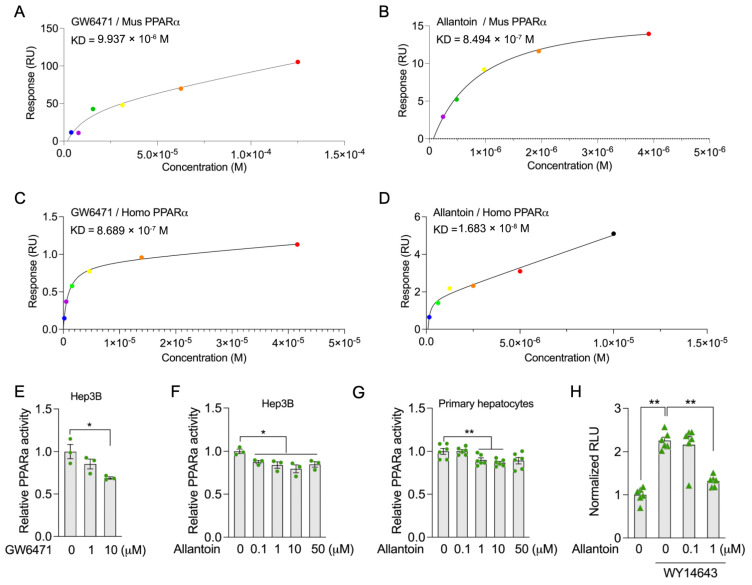
Allantoin is potential endogenous PPARα antagonist. (**A**–**D**) Surface plasmon resonance of allantoin and GW6471, a known PPARα antagonist, with mouse and human PPARα: corresponding curve of GW6471 (**A**) and allantoin (**B**) with mouse PPARα, corresponding curve of GW6471 (**C**) and allantoin (**D**) with human PPARα. (**E**) Relative PPAR activity assay of Hep3B cells with GW6471 treatment for 6 h. (**F**) Relative PPARα activity assay of Hep3B cells with allantoin supplement for 6 h. (**G**) Relative PPARα activity assay of mouse primary hepatocytes with allantoin supplement for 6 h. (**H**) Luciferase reporter assay of PPARα with supplement of allantoin and PPARα agonist WY14643. Values are mean ± SEM, each dot represents one biological replicate. Statistical analysis was conducted using two-tailed unpaired *t*-test. * *p* < 0.05, ** *p* < 0.01.

To test this, purified murine and homo recombinant PPARα proteins were subjected to allantoin binding assays via Surface Plasmon Resonance (SPR). Notably, allantoin presented strong binding affinity with both murine and homo recombinant PPARα proteins, with an equilibrium dissociation constant (Kd) of 0.85 μM and 16.8 nM respectively, which was comparable with GW6471, a known PPARα antagonist (Figure 6A–D). Biochemical analysis using a transcription factor assay kit demonstrates around 30% reduction in PPARα activity after 6 h of GW6471 treatment (Figure 6E). Further analysis of the allantoin supplement demonstrates its ability to suppress PPARα activity in both Hep3B cells and primary hepatocytes (Figure 6F,G). Consistently, luciferase reporter assay further revealed that allantoin could efficiently inhibit PPARα transcription activation by the WY14643, a designed PPARα agonist (Figure 6H).

In addition to PPARα, the nuclear receptor farnesoid X receptor (FXR) is recognized as another regulator of bile acid and lipid metabolism. However, the relationship between PPARα and FXR remains complex, as previous studies have shown both cooperative and antagonistic effects between these two pathways [33]. While FXR activation, such as by the imidazoline I-1 receptor agonist rilmenidine has been shown to ameliorate hepatic steatosis [35]. To investigate whether FXR was involved in mediating allantoin’s effects on hepatic lipids, Hep3B cells were treated with rilmenidine in the presence or absence of allantoin for 24 h. Lipid and cholesterol staining revealed that rilmenidine alone had no significant effects on endogenous TG and TC levels (Figure S7). Furthermore, rilmenidine failed to counteract allantoin-induced lipid and cholesterol accumulation in Hep3B cells (Figure S7), indicating that allantoin’s lipid-modulating effects are independent of FXR signaling. Together with our earlier findings, these results strongly support that allantoin functions as an endogenous antagonist of PPARα, potentially counteracting PPARα activation under diverse stimuli.

## 4. Discussion

Oxidative stress, mediated by ROS, is a key driver of cellular damage and aging, highlighting the critical importance of antioxidant defense systems. Among these, UA, the terminal product of human purine metabolism, occupies a unique position. Unlike most mammals, human purine metabolism lacks functional uricase, an enzyme that oxidizes UA into allantoin, resulting in elevated plasma UA levels [8]. This evolutionary loss was first theorized in the 1980s by Ames et al., who proposed that uricase deficiency conferred an antioxidant advantage in early hominids by enhancing UA’s ability to neutralize ROS like singlet oxygen, peroxyl radicals, and hydroxyl radicals [36]. Subsequent studies further supported UA’s beneficial effects in multiple aspects, especially neuronal protection [37,38]. Paradoxically, despite its antioxidant potential, elevated UA is now implicated in diverse metabolic disorders, including insulin resistance, obesity, T2D, DLP, fatty liver disease, and hypertension, many of which are recognized risk factors for MASLD and known to present systemic oxidative stress [39,40]. Although UA’s chemical structure inherently supports antioxidant activity, its dual role in pathophysiological regulation remains poorly understood. In this study, we identify a mechanistic link, allantoin, the oxidative byproduct of UA often dismissed as biologically inert, drives MASLD progression by disrupting lipid and cholesterol homeostasis via PPARα regulation. This discovery bridges the gap between UA’s antioxidant legacy and its pathological associations, redefining allantoin as a critical mediator of metabolic disorders.

Elevated serum UA level was first reported in patients with MASLD a decade ago [41] and subsequently identified as an independent predictor of MASLD in a case-control study [42]. Ongoing research from China and Western countries has consistently shown that elevated serum UA levels correlate with an increased risk of MASLD [43,44]. However, conflicting results have emerged from a study in Japan involving 2024 participants, which reported different findings [45]. These discrepancies may stem from variations in study criteria and participant demographics. To address this, we conducted an observational analysis involving the largest cohort to date, comprising 80,757 healthy controls, 23,419 individuals with T2D, and 2419 cases of MASLD. Our findings confidently demonstrated a strong correlation between elevated UA and higher levels of TG, increased liver PDFF, and a greater risk of T2D and MASLD, even after adjusting for potential confounders such as sex, age, smoking, and alcohol consumption. However, a further rigorously two-way MR analysis revealed no causal effect between serum UA level and MASLD, which was consistent with the previous study [46]. Notably, Chinese participants with T2D and DLP did not exhibit elevated serum UA levels compared to healthy controls. These findings strongly suggest that UA influences MASLD-related metabolic disorders indirectly.

While humans lost uricase activity during evolution, rendering UA the terminal product of purine catabolism [8], UA can still undergo oxidation to allantoin via ROS. Consequently, allantoin has been widely validated as a clinical marker of oxidative stress [18,19]. Chronic metabolic disorders, such as T2D and DLP, are strongly associated with systemic oxidative stress, which drives persistent elevations in allantoin levels across intracellular and extracellular compartments [47]. Consistent with this paradigm, we observed markedly increased allantoin levels in Chinese individuals with T2D or DLP, underscoring the presence of pronounced oxidative stress in these metabolic conditions. Notably, allantoin, instead of UA, demonstrated a positive correlation with fasting glucose, serum TC, and TG levels in human subjects. This suggests that allantoin, rather than UA, may directly contribute to the pathogenesis of metabolic dysfunction. Subsequent mice studies further revealed that allantoin exacerbates the progression of MASLD, supporting the hypothesis that allantoin mediates the association between UA and risk of MASLD-related metabolic disorders, particularly under stress conditions such as HFD feeding. We propose that under metabolic stress, such as obesity, T2G, or DLP, elevated oxidative activity drives UA oxidation into allantoin, irrespective of baseline UA levels. This allantoin elevation may then accelerate MASLD progression, positioning it as a critical mediator in metabolic pathology.

PPARα has long been recognized as a key regulator of lipid metabolism and is closely involved in the development of MASLD [32]. Although PPARα activity is modulated by diverse metabolite ligands and binding proteins, the complexity of this regulatory network remains incompletely understood. Studies using hepatic PPARα knockout mice have provided clear evidence that hepatic PPARα deficiency promotes the progression of MASLD-related metabolic disorders [48]. Similar observations were made in mice supplemented with allantoin under HFD feeding, which exhibited worsened glucose intolerance, elevated serum TC, and increased hepatic lipid accumulation. These phenotypic changes were accompanied by upregulation of genes associated with lipogenesis and lipid droplet biogenesis. In vitro assays further revealed a strong binding affinity of allantoin with PPARα protein, and suppression of PPARα activity following allantoin supplement, suggesting that allantoin may serve as an endogenous PPARα inhibitor. However, the precise binding characteristics and regulatory mechanisms remain under further investigation. Beyond lipid metabolism, allantoin-supplemented mice under HFD feeding showed significantly reduced bile acid levels, paralleled by a notable negative correlation between allantoin and bile acids in human subjects. Coupled with in vitro evidence of elevated cholesterol levels after allantoin treatment, we hypothesize that allantoin suppresses bile acid biosynthesis, leading to cholesterol accumulation. While recent studies have implicated PPARα in bile acid homeostasis [49,50], the extent to which allantoin influences bile acid levels via PPARα remains unclear and a limitation in the current study. Notably, SPR analysis confirmed allantoin’s binding affinity to PPARα, and its inhibitory effects on PPARα activity raise the possibility of nuclear translocation of allantoin, although the underlying mechanisms remain speculative. While allantoin transport has been partially characterized in non-mammalian systems, such as PucI in Bacillus subtilis [51] and PvUPS1 in French Bean [52], research in mammalian cells remains limited. Notably, prior studies have identified a variety of uric acid transporters in mammals [53], which may provide insights into the mechanisms of allantoin transport. We speculate that specific transporters for allantoin likely exist in mammalian cells, which could play a role in modulating allantoin transport. This intriguing topic certainly merits further investigation.

In summary, this study revealed a robust association between UA levels and MASLD-related metabolic disorders using large-scale UK Biobank data. Mechanistically, UA’s antioxidative properties drive allantoin accumulation through ROS-mediated oxidation under metabolically stressed conditions. We demonstrated that allantoin, a terminal oxidation product of UA, not only serves as a biomarker of oxidative stress in individuals with metabolic disorders but also functions as a risk factor that directly promotes MASLD progression by disrupting PPARα regulated TG and TC metabolism. Collectively, these findings establish allantoin as a critical mediator linking UA to MASLD-related metabolic pathology. This work advances our understanding of UA’s antioxidative role in pathophysiology regulation, and positions allantoin as both a clinical marker and a therapeutic target for managing metabolic diseases.

## Data Availability

The data supporting the findings from this study are available within the article file and its Supplementary Information. The raw transcriptome data that support the findings of this study have been deposited in NCBI Gene Expression Omnibus (accession number GSE283505). To protect the privacy and confidentiality of the participants in human study, personal data including body composition and serum biochemistry data are not made publicly available in a repository or the Supplementary Material of the article. These data are available from the authors (Zhihui Feng) upon reasonable requestion and with permission from the data assessment committee.

## Supplementary Materials

The following supporting information can be downloaded at: https://www.mdpi.com/article/10.3390/antiox14050500/s1, Figure S1: Scatter plot and leave one out analysis plot for the MR analysis; Figure S2: Targeted metabolomics analysis of serum from Chinese elders; Figure S3: Effects of allantoin supplement on mice glucose tolerance; Figure S4. Allantoin increases lipid biogenesis in the liver; Figure S5: Comparative analysis of bile acids level in Chinese elders; Figure S6: Inhibition of PPARα disrupts lipid and cholesterol metabolism; Figure S7. Effect of FXR activation on allantoin-mediated hepatic lipid metabolism; Figure S8. Uncropped gels for Figure S4F. Table S1. Characteristics of participants of urate in UKB; Table S2: GWAS summary-level data; Table S3: The information of IVs for all exposure-outcome pairs; Table S4: Testing for the MR hypotheses; Table S5: Pleiotropy test for significant results of the MR; Table S6: List of primers used in the study. References [54,55,56,57,58,59,60,61,62,63,64,65,66] are cited in the supplementary materials.

## Abbreviations

The following abbreviations are used in this manuscript:

DLP	Dyslipidemia
UA	Uric acid
MASLD	Metabolic dysfunction-associated steatotic liver disease
T2D	Type 2 diabetes
LDL	Low-density lipoprotein
ROS	Reactive oxygen species
PDFF	Proton density fat fraction
TC	Total cholesterol
TG	Triglyceride
PPARα	Peroxisome proliferator-activated receptor alpha
