# Supplementary material for: Allantoin Serves as a Novel Risk Factor for the Progression of MASLD
Source: Antioxidants (Basel). 2025 Apr 22;14(5):500. doi: 10.3390/antiox14050500 (PMC12108491; doi:10.3390/antiox14050500)
Supplement: Supplementary file 1 [file antioxidants-14-00500-s001.zip › antioxidants-3506164-supplementary/antioxidants-3506164 -SM.pdf]

antioxidants

MDPI

Article

Allantoin Serves as a Novel Risk Factor for the Progression of MASLD

Weiqiang Lv [1,2,†], Xueqiang Wang [3,†], Zhaode Feng [1], Cunxiao Sun [2], Hansen Wu [2], Mengqi Zeng [3], Tianlin Gao [4], Ke Cao [1], Jie Xu [1], Xuan Zou [5,6], Tielin Yang [7], Hao Li [1], Lei Chen [3], Jiankang Liu [1,3], Shanshan Dong [7,*] and Zhihui Feng [2,3,8,*]

[1] Center for Mitochondrial Biology and Medicine, The Key Laboratory of Biomedical Information Engineering of Ministry of Education, School of Life Science and Technology, Xi'an Jiaotong University, Xi'an 710049, China; gl824705609@stu.xjtu.edu.cn (W.L.); fengzhaode@stu.xjtu.edu.cn (Z.F.); caoke2016@mail.xjtu.edu.cn (K.C.); xj0322@xjtu.edu.cn (J.X.); lihao@xjtu.edu.cn (H.L.); j.liu@mail.xjtu.edu.cn (J.L.)
[2] Frontier Institute of Science and Technology, Xi'an Jiaotong University, Xi'an 710049, China; 4123128024@stu.xjtu.edu.cn (C.S.); whs740211756@outlook.com (H.W.)
[3] School of Health and Life Sciences, University of Health and Rehabilitation Sciences, Qingdao 266100, China; wangxueqiang@uhrs.edu.cn (X.W.); zengmengqi@uhrs.edu.cn (M.Z.); chenlei@uhrs.edu.cn (L.C.)
[4] School of Public Health, Qingdao University, Qingdao 266071, China; gaotl@qdu.edu.cn
[5] Department of Geriatrics Cardiology, The Second Affiliated Hospital of Xi'an Jiaotong University, Xi'an 710004, China; wooair@gmail.com
[6] Precision Medical Institute, The Second Affiliated Hospital of Xi'an Jiaotong University, Xi'an 710004, China
[7] Biomedical Informatics & Genomics Center, The Key Laboratory of Biomedical Information Engineering of Ministry of Education, School of Life Science and Technology, Xi'an Jiaotong University, Xi'an 710049, China; yangtielin@xjtu.edu.cn (T.Y.)
[8] Interdisciplinary Research Center of Frontier Science and Technology, Xi'an Jiaotong University, Xi'an 710049, China
* Correspondence: dongss@xjtu.edu.cn (S.D.); zhfeng@mail.xjtu.edu.cn (Z.F.)
† These authors contributed equally to this work.

Academic Editor: Alessandra Napolitano

Received: 24 February 2025
Revised: 14 April 2025
Accepted: 18 April 2025
Published: 22 April 2025

Citation: Lv, W.; Wang, X.; Feng, Z.; Sun, C.; Wu, H.; Zeng, M.; Gao, T.; Cao, K.; Xu, J.; Zou, X.; et al. Allantoin Serves as a Novel Risk Factor for the Progression of MASLD. *Antioxidants* **2025**, *14*, x. https://doi.org/10.3390/xxxxx

Copyright: © 2025 by the authors. Submitted for possible open access publication under the terms and conditions of the Creative Commons Attribution (CC BY) license (https://creativecommons.org/licenses/by/4.0/).

1. Supplemental Methods

1.1 Cell culture

Primary hepatocytes: Mice were anesthetized by injecting 0.1 mL of the anesthetic combined solution per 20 g body weight. Perfuse Buffer I (Hanks buffer containing 2 mM ethylene glycol tetraacetic acid and 0.1% glucose, pH 7.4) through the hepatic portal vein, followed by Buffer II (Hanks buffer with 5 mM CaCl2, 0.1% glucose, and 0.6 mg/mL collagenase IV, pH 7.4). Carefully excise the liver and transfer it to a 100-mm cell culture plate containing 10 mL of ice-cold hepatocyte washing medium (HWM). Next, place the liver in ice-cold Hanks buffer and filter it using a 70 μm nylon cell strainer. To disperse the cells, gently blow through the liver tissue with a 25 mL pipette. Allow the cells to rest for 10 minutes, then dilute the settled cells in 20 mL of ice-cold HWM (standard William's Medium E supplemented with 7% v/v FBS, antibiotics, and antifungal agents at a concentration of 10 mL/L) and centrifuge at 50 g for 3 minutes at 4°C. Discard the supernatant and gently resuspend the cells in 20 mL of pre-cooled HWM, washing them through repeated centrifugation. Dispense 1 mL (5 × 10^6 cells/mL) of viable hepatocytes into each well of a Matrigel-coated 12-well culture plate and incubate the cells at 37°C with 5% CO2 for 2–3 hours. Using a pipette, remove the medium along with any dead or unattached cells. Add fresh hepatocyte culture medium (HCM), consisting of standard William's Medium E supplemented with 7% v/v FBS, 10 mg/L insulin, 6.7 μg/L sodium selenite, 5.5 mg/L transferrin, 110 mg/L sodium pyruvate, and an antibiotic/antimycotic solution at 10

mL/L, ensuring a final concentration of 30 mM sodium pyruvate and 5 nM dexamethasone for subsequent experimental work.

Human Hep3B: Cells were obtained from ATCC (Manassas, VA, USA). Hep3B cells were cultured in Minimum Essential Medium (MEM) supplemented with 10% (v/v) fetal bovine serum, along with non-essential amino acids, 2 mM L-glutamine, and 1 mM sodium pyruvate. The culture media was additionally supplemented with 100 U/mL penicillin and 100 μg/mL streptomycin. Cells were incubated at 37 °C in a humidified atmosphere containing 5% CO2, and the culture medium was changed every other day.

1.2 Mendelian Randomization (MR) analysis

Instrumental Variables (IV) selection and quality control: For IV selection, we employed the clumping algorithm within PLINK (https://www.cog-genomics.org/plink/1.9/)[1] to select independent SNPs for the exposure (r^2 threshold = 0.001, window size = 1 Mb and $P < 5 \times 10^{-8}$). We leveraged the 1000 Genomes Project's European data (phase 3) as the reference for linkage disequilibrium (LD) estimation[2]. Subsequently, if a chosen SNP from the initial step was absent from the outcome data, we substituted it with a proxy SNP exhibiting LD $r^2 > 0.8$. The proxy SNP should also be associated with exposure ($P < 5 \times 10^{-8}$). When multiple proxy SNPs met the criteria, we chose the one with the highest LD r^2 and the lowest P value associated with the exposure.

All IVs must hold the three key assumptions of MR analysis: relevance assumption, independence assumption and exclusion restriction assumption. We used RadialMR [3] to remove pleiotropic SNPs. We also looked up each IV and its proxies in the PhenoScanner GWAS database (http://phenoscanner.medschl.cam.ac.uk) [4] to check any possible associations with confounders (smoking and drinking status). The proxy selection was performed by the PhenoScanner database (LD $r^2 > 0.8$ in human GRCh37 genome). SNPs associated with potential confounders were excluded from subsequent MR analysis. We implemented MR Steiger filtering to verify the true causal direction[5].

Two-sample MR analysis: We performed two-sample MR analysis to estimate the causal effects of urate on TG, liver PDFF, MASLD and T2D. We selected the inverse-variance weighted (IVW) method as the primary method for causal inference[6]. To further strengthen the reliability of our findings, we also conducted four other MR methods. The MR-Egger approach estimates the causal effect by using the slope coefficient from the Egger regression, and it could provide a robust estimate even if none of the IVs are invalid[7]. The weighted median method could provide consistent causal effects estimate even when up to 50% of the SNPs are invalid[8]. The weighted mode method yields consistent estimates when the largest number of similar individual-instrument estimates come from valid instruments, even if the majority of instruments are invalid [9]. MR-RAPS accounts for systematic and idiosyncratic pleiotropy and can provide a robust inference with many weak instruments [10]. All these methods were implemented in the TwoSampleMR R package [11].

Sensitivity analyses: For the significant MR results, we performed sensitivity analysis. First, leave-one-out analysis was used to check whether the significant causal associations were driven by a single SNP ($P < 0.05$ was considered as an outlier). Second, we used MR pleiotropy residual sum and outlier (MR-PRESSO) global test [12] to detect horizontal pleiotropy ($P < 0.05$). Third, we also used the intercept term of the MR-Egger method to estimate the directional pleiotropic effect [13] ($P < 0.05$).

1.3 Confocal microscopy

Hep3B cells and primary hepatocytes were seeded onto poly-lysine-coated coverslips and subsequently fixed with 4% paraformaldehyde following the indicated treatments. The coverslips were stained with Nile Red diluted in PBS at a ratio of 1:3000 for 15

minutes, then gently washed three times with PBS. They were further stained with DAPI (Invitrogen, Carlsbad, CA, USA) and imaged using a ZEISS LSM700 fluorescence microscope at 63x magnification (Carl Zeiss, Chicago, IL, USA). Additionally, NBD Cholesterol Staining Dye was added to the cell culture medium and co-incubated with the cells for 2 hours prior to fixation with paraformaldehyde.

1.4 Hematoxylin & eosin (HE) staining

HE staining for morphological evaluation studies were conducted on mouse tissues. Fresh tissues were washed with pre-cooled PBS and fixed in 4% paraformaldehyde (0.1M PBS, pH 7.4) prior to embedding and sectioning. The liver, white adipose tissue, brown adipose tissue, and muscle tissues were cut into 5 μm sections with ultramicrotome for further HE staining following standard protocols.

1.5 RNA sequencing

RNA sequencing of mouse liver samples was conducted by SMART BIOTECH (Tianjin, China). RNA samples were sequenced using an Illumina platform (HiSeq, Illumina, San Diego, CA, USA). The raw data were filtered to generate clean data, which was then used for analysis of KEGG pathways and the creation of volcano plots.

1.6 Protein isolation and western bolting

Liver tissues and cell lysates were prepared using Western and IP lysis buffer (Beyotime, Jiangsu, China), supplemented with 1 mM PMSF. Protein concentrations were determined using a BCA Protein Quantification Kit (Pierce, Rockford, IL, USA). Proteins were then separated by SDS-PAGE electrophoresis and transferred to nitrocellulose (NC) membranes. The membranes were probed with primary antibodies overnight at 4°C, followed by incubation with horseradish peroxidase-conjugated secondary antibodies for 1 hour at room temperature. Western blots were developed using an ECL Western blotting detection kit (Pierce, Rockford, IL, USA) and quantified using scanning densitometry.

1.7 Real-time PCR

Total RNA was extracted from mouse liver tissues or cell samples using TRIzol Reagent (Invitrogen, Carlsbad, CA, USA). The RNA was then reverse-transcribed into cDNA using an RT-PCR kit (TaKaRa, Dalian, China) and subsequently analyzed by semi-quantitative real-time PCR with specific primers. Data were normalized to the mRNA or DNA levels of actin, serving as a housekeeping gene, and analyzed using the $2^{-\Delta\Delta Ct}$ method. The final results are presented as a percentage of the control. The specific primers were provided in Table S6.

2. Supplemental Figures

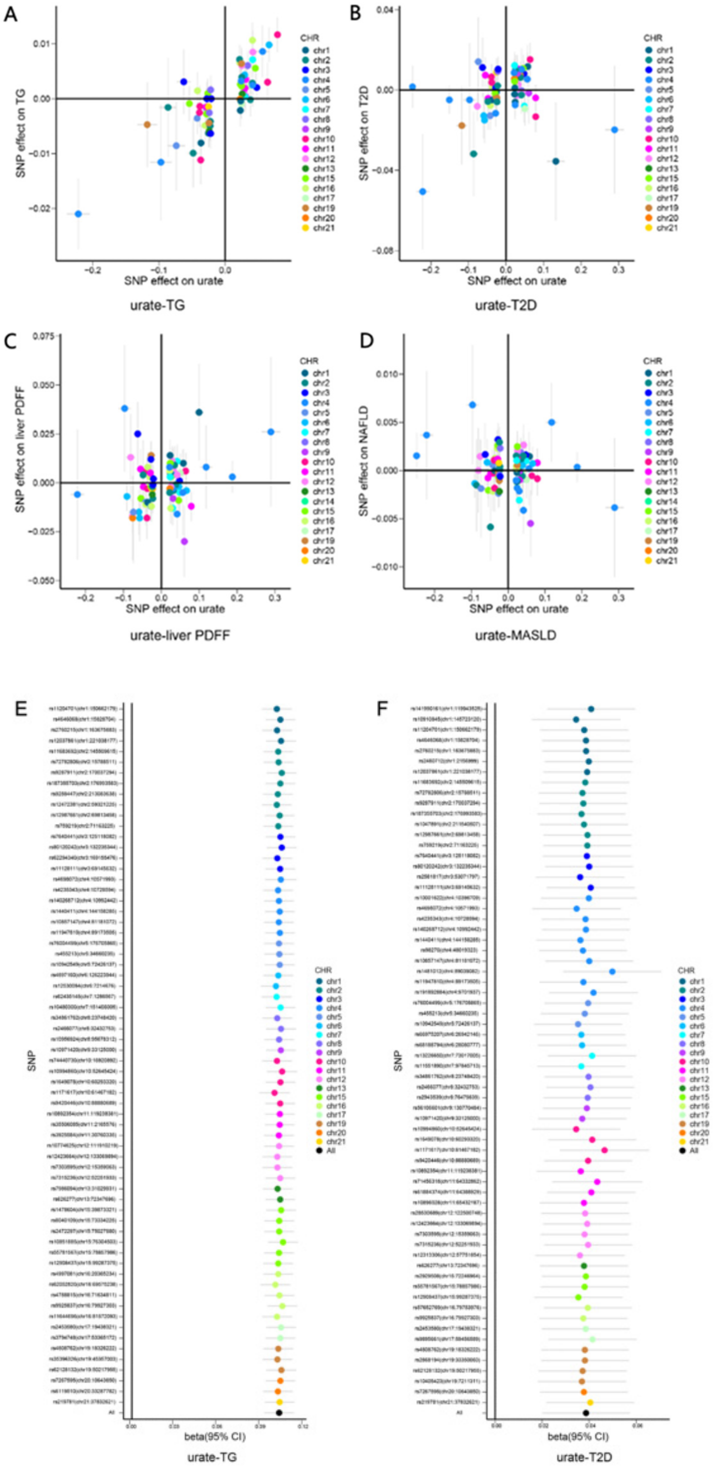

Figure S1. **Scatter plot and leave one out analysis plot for the MR analysis.** (A) Scatter plot of the effect estimates on both urate and TG. (B) Scatter plot of the effect estimates on both urate and T2D. (C) Scatter plot of the effect estimates on both urate and PDFF. (D) Scatter plot of the effect estimates on both urate and MASLD. (E) Leave one out analysis plot of urate against TG. (F) Leave one out analysis plot of urate against T2D.

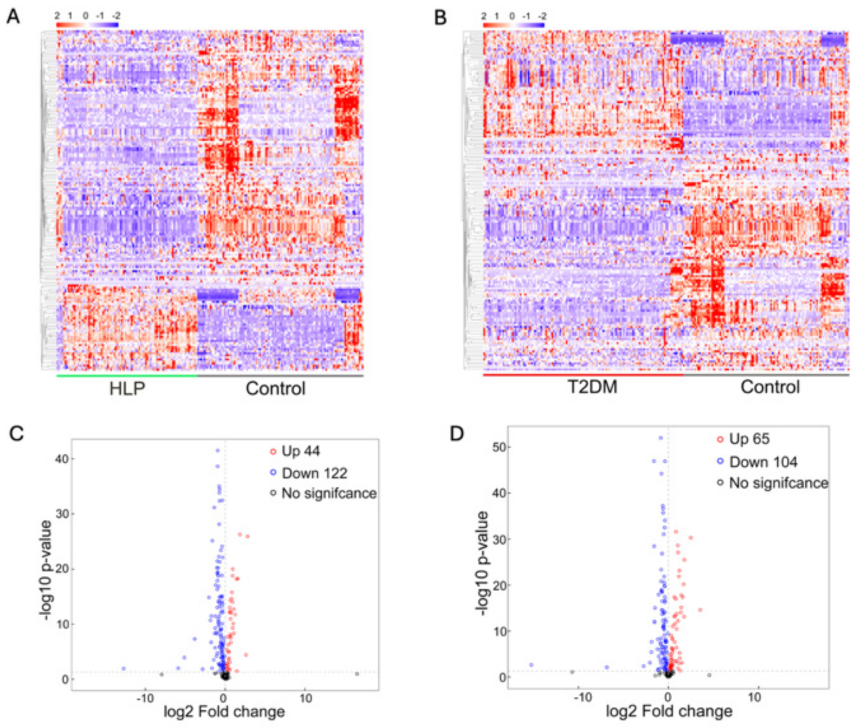

Figure S2. Targeted metabolomics analysis of serum from Chinese elders (A) Hierarchical clustering analysis of identified metabolites in DLP and Control group. (B) Hierarchical clustering analysis of identified metabolites in T2D and Control group. (C) Volcano plot of significant changed metabolites in DLP group comparing to Control group. (D) Volcano plot of significant changed metabolites in T2D group comparing to Control group.

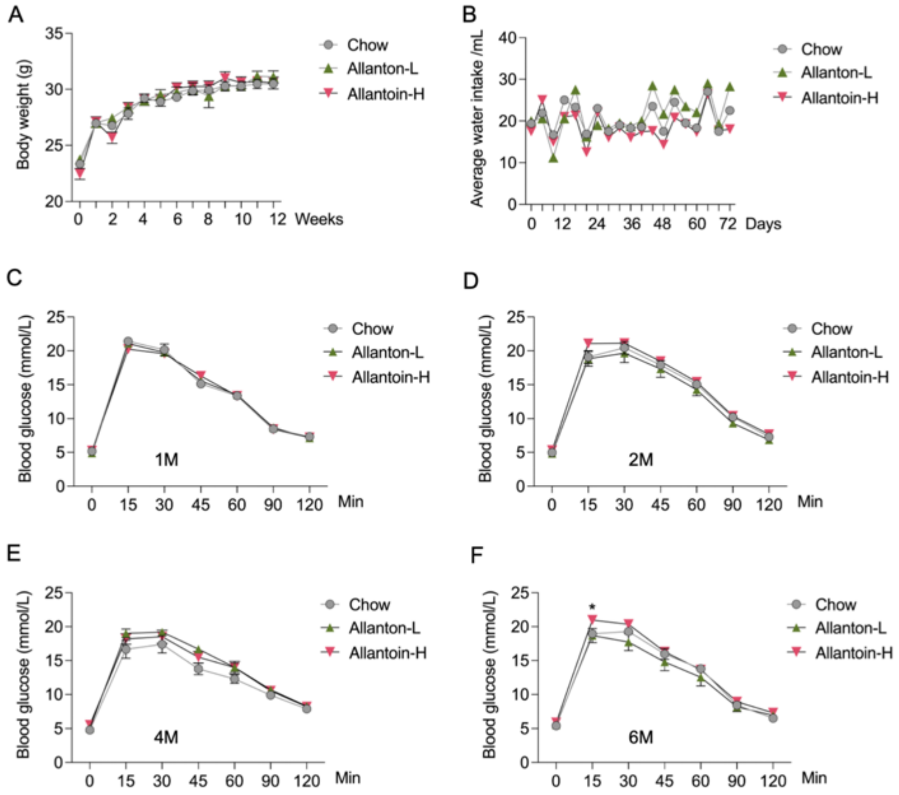

Figure S3. Effects of allantoin supplement on mice glucose tolerance. (A) Body weight curve and (B) average water intake in mice supplement with allantoin for 12 weeks. Glucose tolerance test in mice after allantoin supplement for 4 weeks (C), 8 weeks

(D), 16 weeks (E), and 24 weeks (F). Values are mean ± SEM, $n \geq 7$, each dot represents one biological replicate. Statistical analysis was conducted using two-tailed unpaired *t*-test or two-way ANOVA with multiple comparison test. $^{*}p < 0.05$.

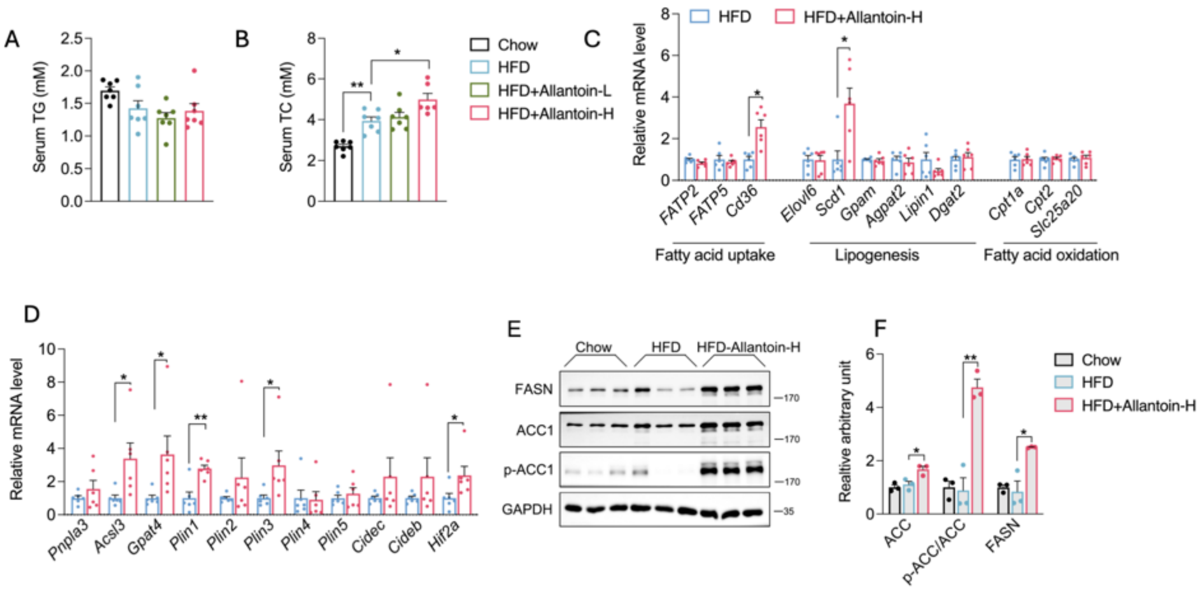

Figure S4. Allantoin increases lipid biogenesis in liver. Mice at age of 8 weeks were subjected to high-fat diet (HFD) for 4 weeks followed by allantoin supplement and HFD feeding for another 4 weeks, (A) serum TG, (B) serum TC level, (C) relative mRNA level of free fatty acid metabolism related genes in the liver, (D) relative mRNA level of lipid droplets biogenesis related genes in the liver, and (E) protein level of FASN and p-ACC1 in the liver was analyzed. Values are mean ± SEM, $n \geq 6$, each dot represents one biological replicate. Statistical analysis was conducted using two-tailed unpaired *t*-test. $^{*}p < 0.05$, $^{**}p < 0.01$.

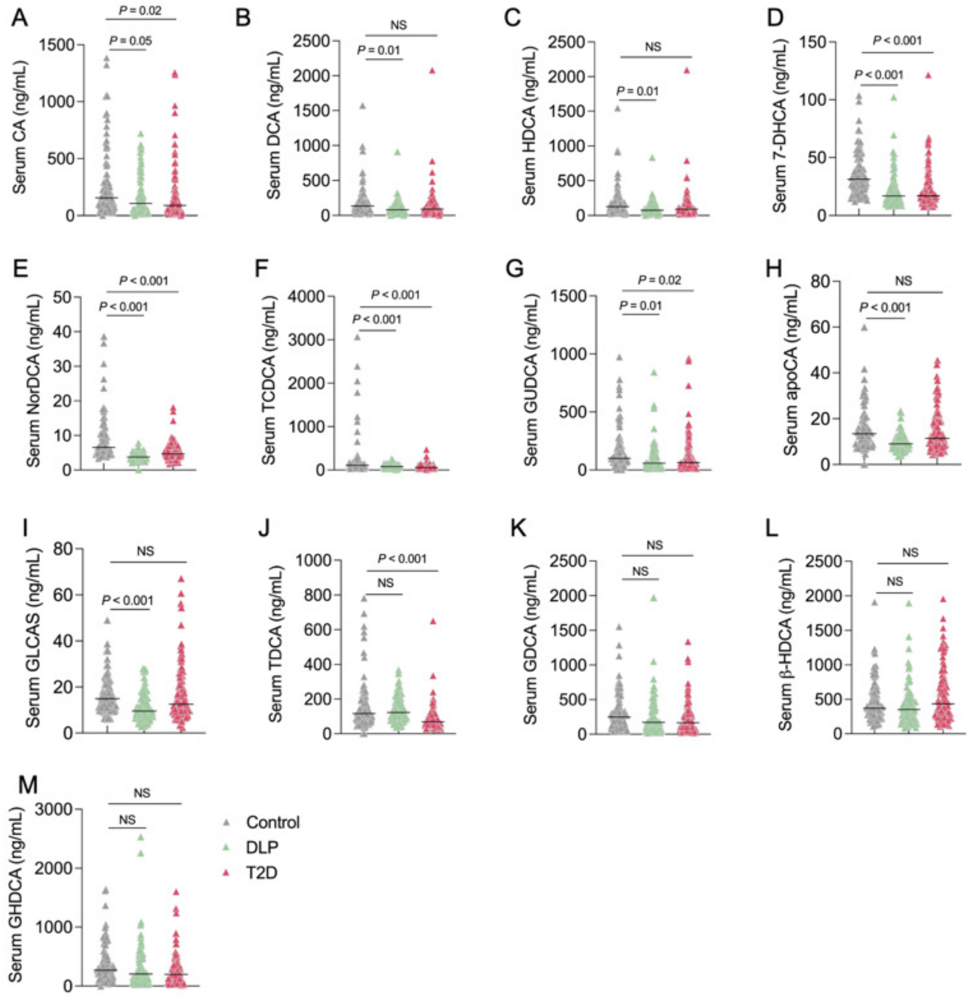

Figure S5. Comparative analysis of bile acids level in Chinese elders. (A) cholic acid (CA). (B) deoxycholic acid (DCA). (C) hyodeoxycholic acid, (HDCA). (D) 7-dehydrocholic acid (7-HDCA). (E) 23-nordeoxycholic acid (NorDCA). (F) taurochenodeoxycholic acid (TCDCA). (G) glycodehydrocholic acid (GUDCA). (H) apocholic acid (apoCA). (I) glycolithocholic acid 3 sulfate (GLCAS). (J) taurodeoxycholic acid (TDCA). (K) glycodeoxycholic acid (GDCA). (L) β-Hyodeoxycholic Acid (β-HDCA). (M) glycohyodeoxycholic acid (GHDCA). Healthy control, n = 105; DLP, n = 89; T2D, n = 126. Values are mean ± SEM, each dot represents one biological replicate. Statistical analysis was conducted using two-tailed unpaired *t*-test.

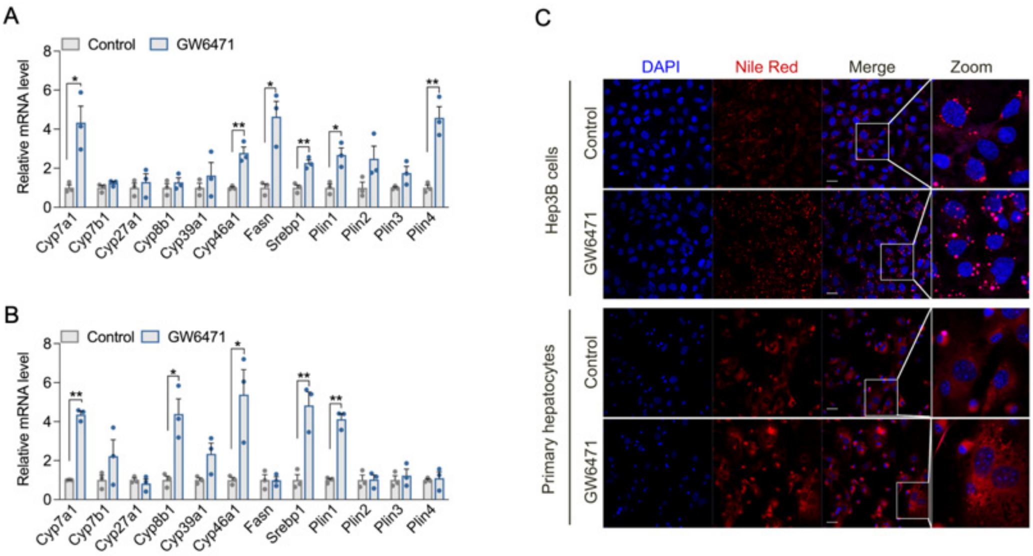

Figure S6. Inhibition of PPARα disrupts lipid and cholesterol metabolism. (A) Relative mRNA level of cholesterol and lipid metabolism related genes in Hep3B cells after GW6471 treatment for 24h. (B) Relative mRNA level of cholesterol and lipid metabolism related genes in primary hepatocytes after GW6471 treatment for 24h. (C) Nile red staining in Hep3B cells and primary hepatocytes after GW6471 treatment for 24h. Values are mean ± SEM, each dot represents one biological replicate. Statistical analysis was conducted using two-tailed unpaired t-test. *$p < 0.05$, **$p < 0.01$.

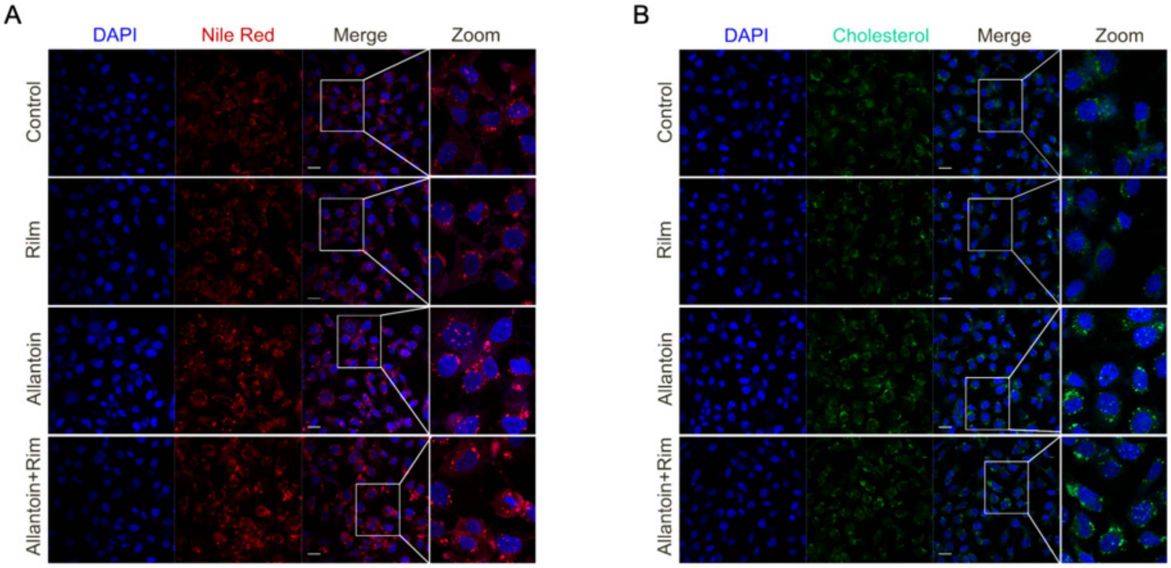

Figure S7. Effect of FXR activation on allantoin-mediated hepatic lipid metabolism. Hep3B cells were pre-treated with rilmenidine at 1μM for 1 h followed by allantoin at 1 μM for another 24 h. Lipid droplets staining (Nile red, A) and cholesterol staining (NBD, B) was then performed (Scale bar, 20 μm).

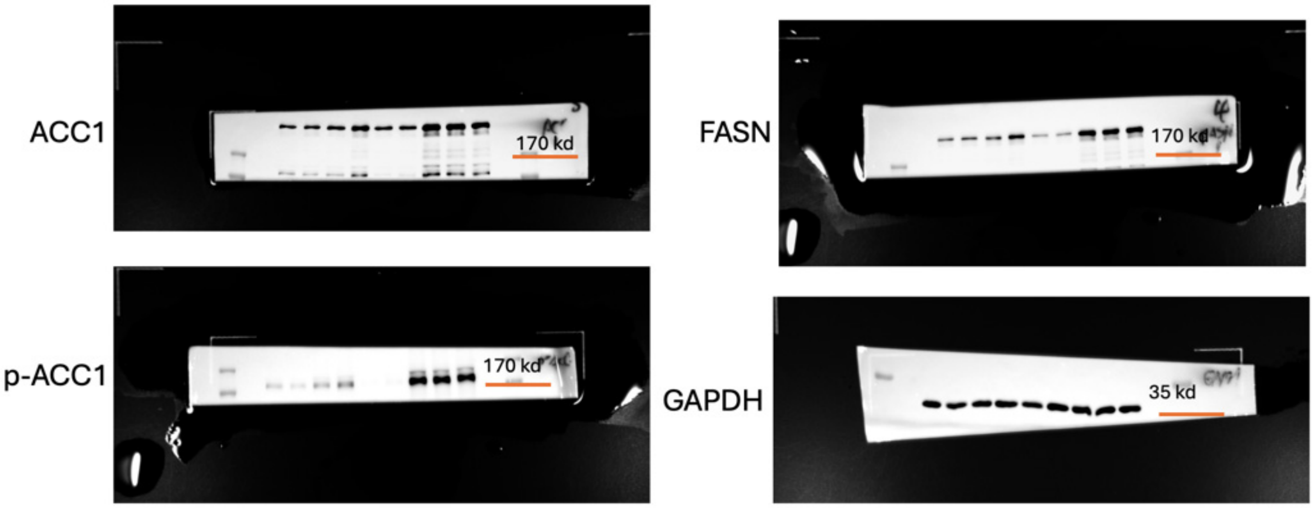

Figure S8. Uncropped gels for Figure S4F

References

1. Purcell, S.; Neale, B.; Todd-Brown, K.; Thomas, L.; Ferreira, M.A.; Bender, D.; Maller, J.; Sklar, P.; de Bakker, P.I.; Daly, M.J., et al. PLINK: a tool set for whole-genome association and population-based linkage analyses. *Am J Hum Genet* **2007**, *81*, 559-575, doi:10.1086/519795.
2. Fairley, S.; Lowy-Gallego, E.; Perry, E.; Flicek, P. The International Genome Sample Resource (IGSR) collection of open human genomic variation resources. *Nucleic Acids Research* **2019**, *48*, D941-D947, doi:10.1093/nar/gkz836.
3. Bowden, J.; Spiller, W.; Del Greco, M.F.; Sheehan, N.; Thompson, J.; Minelli, C.; Davey Smith, G. Improving the visualization, interpretation and analysis of two-sample summary data Mendelian randomization via the Radial plot and Radial regression. *Int J Epidemiol* **2018**, *47*, 2100, doi:10.1093/ije/dyy265.
4. Kamat, M.A.; Blackshaw, J.A.; Young, R.; Surendran, P.; Burgess, S.; Danesh, J.; Butterworth, A.S.; Staley, J.R. PhenoScanner V2: an expanded tool for searching human genotype-phenotype associations. *Bioinformatics* **2019**, *35*, 4851-4853, doi:10.1093/bioinformatics/btz469.
5. Hemani, G.; Tilling, K.; Davey Smith, G. Orienting the causal relationship between imprecisely measured traits using GWAS summary data. *PLoS Genet* **2017**, *13*, e1007081, doi:10.1371/journal.pgen.1007081.
6. Burgess, S.; Butterworth, A.; Thompson, S.G. Mendelian randomization analysis with multiple genetic variants using summarized data. *Genet Epidemiol* **2013**, *37*, 658-665, doi:10.1002/gepi.21758.
7. Bowden, J.; Davey Smith, G.; Burgess, S. Mendelian randomization with invalid instruments: effect estimation and bias detection through Egger regression. *Int J Epidemiol* **2015**, *44*, 512-525, doi:10.1093/ije/dyv080.
8. Bowden, J.; Davey Smith, G.; Haycock, P.C.; Burgess, S. Consistent Estimation in Mendelian Randomization with Some Invalid Instruments Using a Weighted Median Estimator. *Genet Epidemiol* **2016**, *40*, 304-314, doi:10.1002/gepi.21965.
9. Hartwig, F.P.; Davey Smith, G.; Bowden, J. Robust inference in summary data Mendelian randomization via the zero modal pleiotropy assumption. *Int J Epidemiol* **2017**, *46*, 1985-1998, doi:10.1093/ije/dyx102.
10. Zhao, Q.; Wang, J.; Hemani, G.; Bowden, J.; Small, D.S. Statistical inference in two-sample summary-data Mendelian randomization using robust adjusted profile score. *The Annals of Statistics* **2020**, *48*, 1742-1769.
11. Hemani, G.; Zheng, J.; Elsworth, B.; Wade, K.H.; Haberland, V.; Baird, D.; Laurin, C.; Burgess, S.; Bowden, J.; Langdon, R., et al. The MR-Base platform supports systematic causal inference across the human phenome. *Elife* **2018**, *7*, doi:10.7554/eLife.34408.

12. Verbanck, M.; Chen, C.Y.; Neale, B.; Do, R. Detection of widespread horizontal pleiotropy in causal relationships inferred from Mendelian randomization between complex traits and diseases. *Nature genetics* **2018**, *50*, 693-698, doi:10.1038/s41588-018-0099-7.
13. Hemani, G.; Bowden, J.; Davey Smith, G. Evaluating the potential role of pleiotropy in Mendelian randomization studies. *Human molecular genetics* **2018**, *27*, R195-r208, doi:10.1093/hmg/ddy163.

Disclaimer/Publisher's Note: The statements, opinions and data contained in all publications are solely those of the individual author(s) and contributor(s) and not of MDPI and/or the editor(s). MDPI and/or the editor(s) disclaim responsibility for any injury to people or property resulting from any ideas, methods, instructions or products referred to in the content.
